# Telemedicine as a tool for continuing medical education

**DOI:** 10.1093/fampra/cmad085

**Published:** 2023-08-14

**Authors:** Ana Rita J Maria, Helena Serra, Maria G Castro, Bruno Heleno

**Affiliations:** Regional Health Administration of Lisbon and Tagus Valley; Comprehensive Health Research Centre (CHRC), Nova Medical School | Faculdade de Ciências Médicas, Universidade NOVA de Lisboa, Lisbon, Portugal; Interdisciplinary Centre of Social Sciences (CICS. NOVA), NOVA School of Social Sciences and Humanities | Faculdade de Ciências Sociais e Humanas, Universidade NOVA de Lisboa, Lisbon, Portugal; Cardiology Department, Centro Hospitalar e Universitário de Coimbra (CHUC), Coimbra, Portugal; Regional Health Administration of Lisbon and Tagus Valley; Comprehensive Health Research Centre (CHRC), Nova Medical School | Faculdade de Ciências Médicas, Universidade NOVA de Lisboa, Lisbon, Portugal

**Keywords:** continuing medical education, continuity of care, health information, multidisciplinary care, primary care, qualitative research, telemedicine

## Abstract

**Background:**

There is a growing interest in the use of digital technologies to foster learning in the health professions, along with the drive to expand teleconsultations arising from the COVID-19 pandemic. This study aims to explore whether telemedicine between levels of care can act as continuous medical education (CME) tool for general practitioners (GPs) and hospital consultants at the referral cardiology department.

**Methods:**

This qualitative study was embedded in an organizational case study of the introduction of a new service model in the Portuguese health system. Semi-structured interviews were audio-recorded and pseudonymized. The transcribed interviews were stored, coded, and content analysis was performed in MAXQDA.

**Results:**

A total of 11 physicians were interviewed. GPs and cardiologists recognized that telemedicine between levels of care could act as a CME tool. Although they departed with different expectations, telemedicine helped them collaborate as a multidisciplinary team, exchanging feedback about clinical decisions, and constructing knowledge collaboratively. Telemedicine also supplemented existing learning meetings. The consequences of technology adoption may be viewed as a result of the actors involved (including the technology itself), characteristics of the context (including the organization), and an interaction between such factors.

**Conclusion:**

Teleconsultations can be a learning opportunity for the health professionals involved. Our findings suggest that, in the context of the Portuguese health system, telemedicine as a CME tool helped to build multidisciplinary teams which exchanged feedback and constructed shared knowledge to improve patients’ outcomes. It also helped to identify practice-changing contents to be included in face-to-face educational meetings.

**Key messages**

- Physicians felt the collaboration is especially needed when questions occurred.
- Telemedicine helped to construct shared knowledge between levels of care.
- Impact on physicians’ administrative tasks should not be underestimated.
- Participants’ perception shape the implementation process of teleconsultations.

## Background

There is a growing interest in the use of digital technologies to foster learning in the health professions. A systematic review concluded that telelearning technologies achieve comparable learning outcomes to traditional face-to-face lectures.^1^ A challenge remains on how to use technologies to foster reflection on professional practice and making changes to this practice to reduce gaps in performance, which is a key role of continuous medical education (CME).^2^

Teleconsultations can be a learning opportunity for the health professionals involved. Studies have explored the impact of teleconsultations on CME and learning by physicians. Both store-and-forward^3^ and real-time^4^ teleconsultations have been associated with increased learning. The understanding is that due to the active participation of general practitioners (GPs) in real-time teleconsultations, they feel less isolated from peers.^5^ Through teleconsultations, GPs are better at identifying patients who need secondary care and those that can be managed in primary care.^6^ Teleconsultations appear to lead to knowledge transfer from secondary care specialists to GPs,^7,8^ which could have an impact in the health care quality overall and the referral rates. Besides, distant education via telemedicine and the concept of sharing expertise from academic centres, has shown a good option to improve the exposure of health professionals to complex cases and help in their decision-making process.^9^

Our previous work explored physicians’ and patients’ perspectives on the adoption of teleconsultations between primary care and the referral cardiology department.^10^ This study aims to explore whether telemedicine between levels of care can act as a CME tool for GPs and hospital consultants.

## Methods

This qualitative study was embedded in an organizational case study of the introduction and rollout of a new service model. This single case study design^11^ was used to obtain an in-depth understanding of the participants’ perception of a teleconsultation intervention. This study design captures phenomenons that are time-context-dependent and influenced by the individual’s experience and characteristics.^11^ We chose qualitative methods because they help to identify unexpected negative experiences and provide in-depth understanding of how participants’ interactions with interventions produce change.^12^ COREQ^13^ checklist was used to structure and report the study ﬁndings, as referred by enhancing the quality and transparency of health research network.

### Setting

This study occurred before the COVID-19 pandemic, at a time when the Portuguese national health system (NHS) was seeking to made teleconsultations available. It took place in 6 primary care outpatient clinics and the Cardiology department of the Hospital and University Center of Coimbra-General Hospital. This referral hospital is located at the Central Regional Health Administration, where approximately 1.6 million people live.

In the Portuguese NHS, GPs act as ‘gatekeepers’ to secondary care. GPs refer patients through a store-and-forward digital platform. A senior hospital consultant triages requests through the platform and can schedule a hospital appointment or decline the referral and provide feedback to the GP instead. Communication from the hospital to primary care relies on paper letters written from the consultant to the GP or through remote access from the GP to the hospital electronic health record.

This study assesses the implementation of an alternative referral pathway. Instead of asking for an hospital appointment, the GP could refer the patient to a joint teleconsultation of the GP, cardiologist, and the patient. This teleconsultation happened through videoconferencing between the primary care clinic (where GP, patient, and caregiver were located) and cardiology clinic (where the cardiologist was located). The teleconsultations were implemented for both first referrals and follow-up. No remote monitoring of patients was implemented in this project.

We included physicians that participated in at least 1 teleconsultation for real-time communication when requesting or providing patient-specific information. We excluded physicians who are unable or unwilling to give informed consent for participation or who withdrew at any point of the data collection. All the physicians who were participating in this pilot project were included; no physicians refused to participate or dropped out. Participants were interviewed at the primary care practice (GPs) or at the cardiology department (cardiologists). All interviews were conducted by Ana Maria , who is an MD working as a GP (in a practice not involved in this study), and a PhD student, whose research focuses on telemedicine, with training on qualitative methods research and experience conducting focus groups. No one else was present during the interview besides the participants and the first author. The physicians interviewed knew that the first author was a GP working at a distant setting, without previous experience in teleconsultations within clinical practice. One of the physicians interviewed was known by the first author, as he is a coadviser of her PhD thesis. Data saturation was discussed with HS, a medical sociologist, her research focuses on qualitative methods and health care organizations.

### Data collection and data analysis

Informed consent was obtained prior to the participation in any study activity. A semi-structured interview guide was developed based on a review of the literature and discussions among the authors (Supplementary Material). The questions composing the interview guide were pilot-tested with patients and physicians outside the study setting. We reflected on the dynamics and process of introducing teleconsultations into health care with 2 of the most frequently used theories in telemedicine research,^14^ namely, normalization process theory,^15,16^ and the diffusion of innovations,^17^ although there is scarce integration of constructs belonging to different theories found in the literature. Both of these theories highlight that the ongoing use of technology is linked to flexibility to enable care models to meet end-users’ needs and expectations, which in turn will evolve as new challenges appear.

No repeated interviews were conducted. Field notes were made during the interviews, which were audio-recorded and pseudonymized. The digital audio recordings were transcribed verbatim by an external transcribing service, and the transcripts were validated by the interviewer against the recorded material. With the advent of the COVID-19 pandemic, transcripts and findings were not returned to the participants for comments, which was revealed to be a strenuous and time-consuming task for physicians.

All transcribed interviews were analysed independently by the researchers to improve reliability. The transcripts were stored, coded, and analysed in MAXQDA (software for qualitative data analysis, 1995–2016, VERBI Software-Consult Sozialforschung GmbH, Version 12.2.1, Berlin, Germany).

Data analysis followed the steps for conventional content analysis.^18^ The interviewer and 1 other researcher coded all data, any discrepancies were discussed until consensus was reached. The coding system was refined until no further codes emerged. After discussion with other team members, codes were categorized. We conducted an open, inductive analysis, starting with open coding. As the objectives of the study are primarily contextual, concerned with understanding people’s experiences, attitudes, and the nature of the system, the inductive approach keeps a close focus on people’s experiences and ideas.

Other ways of thinking critically about data analysis were also be employed, including comprehensive data treatment and deviant case analysis. This involved a re-interrogation of the data searching for new themes not covered by the initial data analysis.^19^ We looked for deviant data to contradict our emerging analysis, a process known to add validity.^19^ To ensure conﬁdentiality, interviews were numbered sequentially and the participants’ quotes within findings were identified by the respective numerical code.

## Results

We interviewed 11 physicians between September 2019 and January 2020 (Table 1) and found 5 main themes (Table 2). The interviews lasted a total of approximately 5 h (mean 27 min).

**Table 1.** Characteristics of study physicians.

	Number	Female/Male	Urban/Rural practices	<5 years in practice	5 to 20 years in practice	>20 years in practice
GP	7	6/1	¾	1	5	1
Cardiologist	4	2/2	4/0	1	0	3

**Table 2.** Themes related with CME during the implementation of teleconsultations.

Participants’ educational expectations
Feedback exchange
Knowledge construction
Learning meetings
Multidisciplinary health care teams

### Participants’ educational expectations

Our interviews provide data about clinicians’ educational perceptions of joint teleconsultations. We found that hospital consultants expect the GPs to present the patients’ history and the problem to be addressed, while GPs expect solutions regarding diagnosis and management as well as justification for the course of action.

GP5: we know the patient’s family context, we know the patient from another perspective, focussing not only on the disease but the patient as a whole. Sometimes it’s not possible to write all the information and colleagues will treat a problem, and it can happen, not seeing that problem is also at the base of others. In the example of cardiology, they focus on the cardiac part, but then we have to watch the rest and we want to understand what explains the clinical findings.

Cardiologists (C) 4: we often forget that the patient is a whole… We want to start anticoagulants following the guidelines, but then, maybe it’s not such a good idea. During the consultation, something may be unnoticed, because the patient does not know how to tell us. The family doctor in the background knows everything.

### Feedback exchange

GPs want to increase their professional knowledge for the benefit of their patients and that seems to be the main reason why they support the development of new collaborative practices with hospital consultants.

GP7: for us doctors, it is a support to clarify our doubts and our insecurities. For the patients it’s obvious that it’s great because if I have a question and I clarify it, for them, it’s great too.

We always end up learning with the interaction between different colleagues. Although I think that afterwards there must always be something more structured, we cannot learn only from our personal experience or from the experience of our colleagues. We also have to learn in a different way, it’s not just because of what we see others doing.

Feedback exchanged was positively appreciated in both directions. The shared understanding of a problem and its solution is connected with the learning potential between the 2 levels of care, which could contribute significantly to continuity of care and patient safety. As a result of a collaborative practice through the teleconsultation service, GPs have access to advice from cardiologists, which results in an additional course of action being undertaken and a reduced need for face-to-face hospital visits.

C1: patients come to us, we take care of them, we anticoagulate and that’s it, then the patient is gone. We lose track of them. A long-term feedback I think could be very important, for us to have an idea of what is going on in the real world. It is an added value of this whole process.

GP2: my experience was very positive. I think it was also a good opportunity for learning and it was, this was the great advantage that I got from teleconsultation. There are situations in which we have some doubts about whether or not to send it to Cardiology, some exams that have somewhat dubious results, we do not know very well how to guide and this made the knowledge updating possible.

### Knowledge construction

Through experience, clinicians developed know-how that they were able to gather when managing similar cases in the future. GPs valued the unique opportunities to learn and to conﬁrm their course of action with reassurance from the dialogue with hospital consultants that teleconsultations enabled. Also, the cardiologists recognized that teleconsultations helped them to construct shared knowledge, as a direct product of system features that enabled synchronous communication between the parties.

GP4: we learned a lot, we also enriched personally because we learned for that 1 [*patient*] and for others, because there are similar cases and we cleared our doubts.

C2: while meeting the needs of each patient, we are approaching and defining ourselves, we are also learning, this is a learning curve for everyone. I think there is always room for learning both on our part and on the part of the family doctors.

C3: many times it probably starts in 1 case, they have 2 cases for example to discuss and we finish, they then say ‘can we discuss more, just 1 or 2 or 3 more questions?’, and this conversation turns out to be a bit informal.

### Learning meetings

The interaction during the teleconsultations may promote the identification of learning needs. Both groups of physicians also valued the learning meetings promoted regularly between hospital services and the primary care units, where GPs and hospital consultants meet face-to-face in order to discuss the co-management of commonly seen conditions. It is suggested that clinical guidelines and best practices could also be integrated as point-of-care CME for common clinical questions.

C1: we already have the Family Medicine meeting usually in May. I’ve already done the program, I’m now waiting for feedback. The themes that were selected were themes that were proposed by the GPs, and so, these are very interesting topics.

GP2: there’s always new stuff coming out and we can’t keep up with all areas. And so, if we are exchanging ideas, with colleagues from various specialties, it is much easier for us, this learning is much easier. Maybe a cardiology colleague has the part of, his updates, but then there are updates, besides cardiology there is dermatology, there are updates after nephrology, there are… we ended up with a series of things and it’s important also if they help us select what is most important, I think so.

### Multidisciplinary health care teams

Our results reflect that while health service provision is becoming more interdisciplinary, specialized health advice today comes from many more sources. Telemedicine helps clinicians of different specialties at different organizations to learn together to better solve patients’ problems. Ultimately, the cooperation may be not only cross-professional but also cross-organizational.

C4: the relationship that I think is fundamental and that telemedicine allows is a multidisciplinary discussion with the patient, in real time, in which we often have all the patient’s problems. There can be a multidisciplinary team to solve the question, the patient’s problem.

Because sometimes ‘let’s leave it [*the problem*] in the pocket to solve it later’. And I think that, above all, the teleconsultation allows us to solve the problem in real time, that many times by calling we can solve the problem, which is fundamental for the patient and for us.

## Discussion

GPs and cardiologists recognized that telemedicine between levels of care could act as a CME tool. Although they departed with different expectations, telemedicine helped them collaborate as a multidisciplinary team, exchanging feedback about clinical decisions, and constructing knowledge collaboratively. Telemedicine also supplemented existing learning meetings, where the GPs and the cardiologists of the referral hospital can connect. However, to the best of our knowledge, there was no previous personal relationships between the physicians before the project started. Our previous work^20^ found that GPs and cardiologists expressed positive attitudes to the development of professional relationship between them, describing the advantages of the interactive learning process. However, our analysis elucidated the ways in which joint teleconsultations created interactional difficulties in practice. Besides, the professional duty of providing care prevails in minds of care providers and takes precedence over dealing with administrative coordination of care.^20^

New collaboration models such as teleconsultations may be a way to acquire knowledge.^21–24^ Studies assessing joint consultations have shown that this collaboration results in better clinical skills and fewer referrals without any apparent negative effects on patients.^25–27^ Our data show that teleconsultations may improve medical decision making. It also illustrates that, through telemedicine, expertise can move to a different organization without the need for patient or the doctor having to physically move.^23^

Working together, clinicians develop an equivalent to the brainstorming phase in problem-based learning. This process has 2 main advantages. First, it helps to ensure that connections are made between the new information and previous knowledge. Second, it broadens the range of differential diagnosis and requires clinicians to explain their reasoning, which are important skills when healthcare professionals are faced with complex patients.^28^ In this study, the synchronous communication between the parties allowed for immediate feedback. Educational value has also been reported for asynchronous communication^29,30^ in which GPs were able to gain knowledge when a new or additional course of action was suggested by the hospital consultant. Our data show that learning was bidirectional, as cardiologists recognized that the GP knows the patients’ background in a way that impacted the decision-making process. Regular technology-mediated conversations along with face-to-face sessions may allow a balanced solution for building trust between professionals if they are physically distant.^31^

Our previous work described how physicians perceived that teleconsultations prevented the occurrence of low-value specialty visits due to incomplete workups and referrals for problems that could be managed in primary care.^32^ Also, there were cases where the GP had chosen not to refer the patient, but the cardiologist suggested referral after the teleconsultation as earlier access to specialty care could result in quicker treatment.^32^ Additionally, we found that teleconsultations may contribute to improved coordination of care because of expedited communication between GPs and cardiologists. Physicians discussed that real-time interaction had advantages over the existing asynchronous technological interface.^32^

The more diverse a learning group is, the more likely the individuals within the group are to learn.^27^ Telemedicine can be used for multidisciplinary clinical meetings,^33^ translating into both cross-professional and cross-organizational cooperation. The patient can also help physicians reflect on knowledge learned from practice-based consultation, as this is a potential opportunity for CME.^29^ In a group that is working well, other group members will pose questions and seek clarification, which might lead to co-construction of knowledge.^28^ Further research involving telemedicine within multidisciplinary teams may explore the different skills and perspectives that can be integrated and result in a broader total understanding of the problem. Encouraging discussion and reflection will increase learning opportunities which require time and a structure, if they are to be properly integrated into the system.

### Strengths

To the best of our knowledge, it is the first in-depth description of the implementation of teleconsultations in Portugal. As the volume of teleconsultations increases, the range of CME strategies between levels of care require a broad assessment of the way that care processes are structured. Our findings provide information for other health systems seeking to design teleconsultations to help streamline patient care.

Interviews were conducted by 1 researcher using an interview guide, which favours the consistency of questioning and rapport with participants. Interviewing both experienced and younger physicians working in a city or in rural districts makes our results more valid, as the CME process relies on individual and contextual characteristics. Discussions by the analysis team, comprised of both clinical and nonclinical researchers, with different backgrounds at the educational level, facilitated consideration of alternate interpretations of ﬁndings.

### Weaknesses

The online CME is quite distinct from the traditional (in-person) approach and our findings might not capture all the differences, focussing the teleconsultation experience. Our previous work described technical issues using the system, as the communication may become less effective, which might adversely affect the experience of learning.^10^ It is also possible that teleconsultations nonadopter clinicians have different opinions compared to those who participated in our study.

Although the themes seemed to be consistent across the health care units, they have distinct organizational characteristics, within different clinical settings, and the local impact of these aspects may have been underappreciated, for example limitations in areas with poor internet connectivity/low bandwidth. Despite the Portuguese NHS having a nationwide accessible electronic medical records network at the primary-secondary care interface, many regions currently do not support readily shareable information across different health care sites. As a result, our findings may not reflect co-management practices and attitudes in all the NHS medical centres (public health care institutions) and in private practice. Finally, the data used in this analysis is from the perspective of GPs and cardiologists, but knowledge and attitudes regarding CME through teleconsultations from different specialities might differ.

### Future studies

Further research is required to assess the impact of teleconsultations on process variables (including the quality of the referral process and of cross-organization communication) and on patients’ clinical outcomes. Participatory design strategies, which include clinicians as key players in the development and implementation of teleconsultations, may improve the quality of interventions and promote their broader implementation.

### Conclusions

Teleconsultations between levels of care can act as a CME tool. The consequences of technology adoption do not consist of a unique cause-effect relationship, but rather may be viewed as a result of the actors involved (including the technology itself), characteristics of the context (including the organization), and an interaction between such factors. Our findings suggest that, in the context of the Portuguese NHS, telemedicine as a CME tool helped to build multidisciplinary teams which exchanged feedback and constructed shared knowledge to improve patients’ outcomes. It also helped to identify practice-changing contents to be included in face-to-face educational meetings. Adequate organizational support and financial support are necessary for this new collaborative practice to succeed. Identifying specific and recurring clinical content that is most behaviour changing may help guide continuing professional development among physicians.

## Supplementary Material

cmad085_suppl_Supplementary_Material_1

cmad085_suppl_Supplementary_Material_2

cmad085_suppl_Supplementary_Checklist

## Data Availability

An aggregate summary of the data generated during this study is included in this published article. Individual data transcripts cannot be shared publicly due to confidentiality. The datasets used and/or analysed during the current study are available from the corresponding author on reasonable request.
