# Supplementary material for: Telemedicine as a tool for continuing medical education
Source: Fam Pract. 2023 Aug 14;40(4):569–74. doi: 10.1093/fampra/cmad085 (PMC10667068; doi:10.1093/fampra/cmad085)
Supplement: cmad085_suppl_Supplementary_Material_1 [file cmad085_suppl_supplementary_material_1.docx]

## Guide for interviewing physicians

Thank you for agreeing to participate. Let's now start the interview.

We are interested to know your experience with teleconsultations. Please let me know at any time if you need any clarification or if you are not comfortable answering a question. I will start recording now.

1. How was your experience as a doctor in teleconsultation with a colleague?

2. How can a teleconsultation make a difference for your patients?

3. How can a teleconsultation make a difference in the coordination with GPs/ cardiologists?

4. What are the advantages and disadvantages of the teleconsultation?

5. How does a teleconsultation influence your relationship with the patient and with your colleagues?

6. How does a teleconsultation compare to a face-to-face consultation in terms of quality and effectiveness of healthcare?

7. Usually, without teleconsultation, how do you feel the process of coordination between primary health care and secondary health care?

8. Can you remember an example where this coordination between levels of care went well? If yes - what do you consider went well?

9. Can you remember an example where this coordination between levels of care went less well? If yes - what was the problem?

10. How could this problem you mentioned be overcome?

11. How to integrate the patient's needs into the coordination between levels of care?

12. How do you see the possibility of continuing medical education through interaction with your colleagues?

13. Is there anything you would like to add?

Before I finish, I have the last brief questions:

- How many years have you been a doctor?

- How large is your patient list? (to the GP only)

- What is the volume of consultations you usually do per month/year? How many inpatients? (to the cardiologists only)

I will stop recording now. Thank you so much for your time today. Please feel free to contact me at any time if you have any questions or comments about the study.
