# Supplementary figures and images for: Telemedicine as a tool for continuing medical education
Source: Fam Pract. 2023 Aug 14;40(4):569–74. doi: 10.1093/fampra/cmad085 (PMC10667068; doi:10.1093/fampra/cmad085)

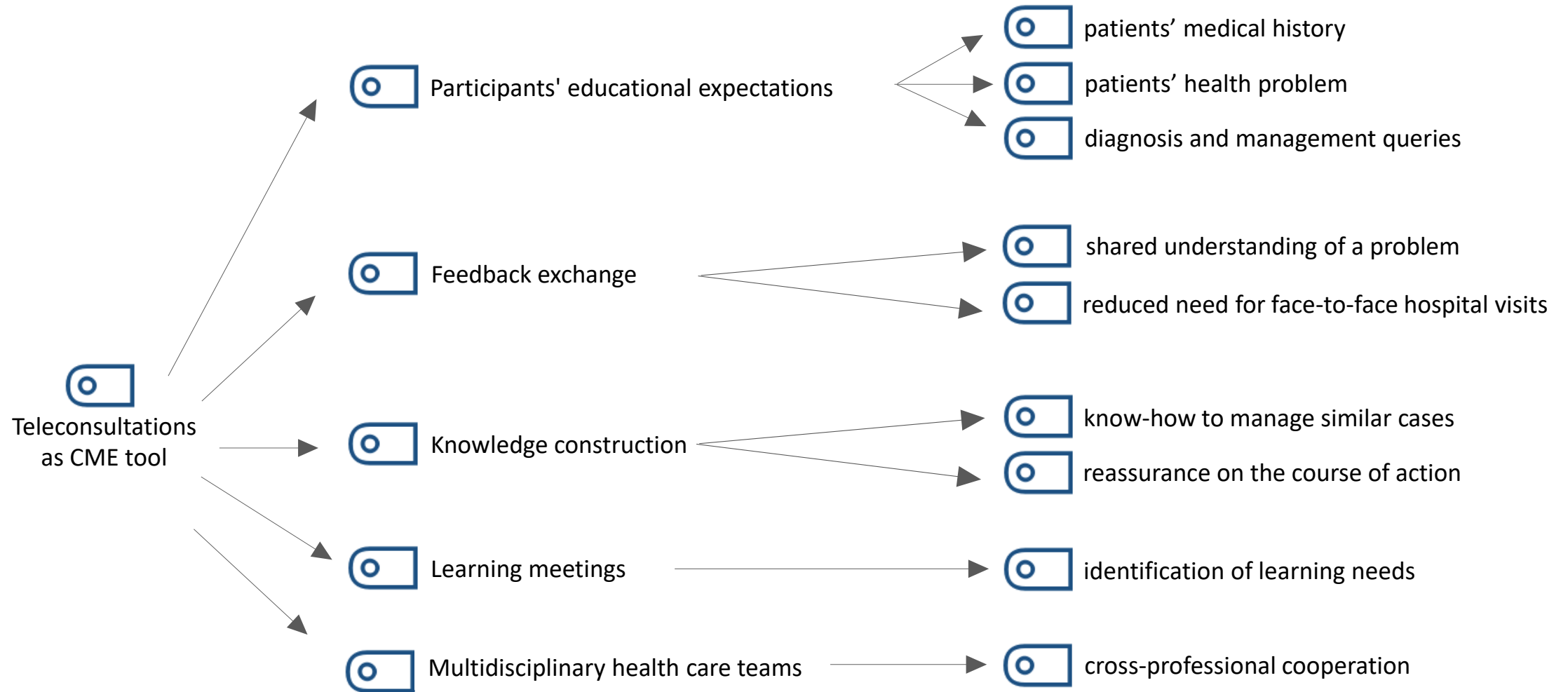

Supplement: cmad085_suppl_Supplementary_Material_2 [file cmad085_suppl_supplementary_material_2.pdf]
